# Implementing a Childhood Cancer Outcomes Surveillance System Within a Population-Based Cancer Registry

**DOI:** 10.1200/JGO.17.00193

**Published:** 2018-03-07

**Authors:** Oscar Ramirez, Paula Aristizabal, Alia Zaidi, Raul C. Ribeiro, Luis E. Bravo, on behalf of VIGICANCER Working Group

**Affiliations:** **Oscar Ramirez** and **Luis E. Bravo**, Universidad del Valle; **Oscar Ramirez**, POHEMA (Pediatric Oncologist and Hemotologist) Foundation, and Centro Médico Imbanaco, Cali, Colombia; **Paula Aristizabal**, University of California San Diego/Peckham Center for Cancer and Blood Disorders, Rady Children’s Hospital, San Diego, and University of California San Diego Moores Cancer Center, La Jolla, CA; and **Alia Zaidi** and **Raul C. Ribeiro**, St Jude Children’s Research Hospital, Memphis, TN.

## Abstract

**Purpose:**

Approximately 80% of cases of childhood cancer occur in low- and middle-income countries and are associated with high mortality rates. Assessing outcomes is essential for designing effective strategies to improve outcomes equally worldwide. We implemented a real-time surveillance system, VIGICANCER, embedded in a population-based cancer registry (PBCR) to assess childhood cancer outcomes.

**Methods:**

VIGICANCER was established in 2009 as an integral part of Cali’s PBCR to collect real-time data on outcomes of patients (age < 19 years) with a new diagnosis of cancer treated in pediatric oncology units in Cali, Colombia. Baseline and follow-up data (death, relapse, treatment abandonment, second neoplasms) were collected from medical records, hospital discharge logs, pathology reports, death certificates, and the National Public Health Insurance database. A quality assurance process was implemented for the system.

**Results:**

From 2009 to 2013, data from 1,242 patients were included in VIGICANCER: 32% of patients were younger than 5 years, 55% were male, and 15% were Afro-descendants. International Classification of Childhood Cancer group I diagnoses predominated in all age groups except children younger than 1 year old, in whom CNS tumors predominated. Five-year overall survival for all cancers was 51.7% (95% CI, 47.9% to 55.4%) for children (< 15 years), and 39.4% (95% CI, 29.8% to 50.5%) for adolescents (15 to 18.9 years). Five-year overall survival for acute lymphoblastic leukemia was 55.6% (95% CI, 48.5% to 62.2%).

**Conclusion:**

Our study demonstrates the feasibility of implementing a real-time childhood cancer outcomes surveillance system embedded in a PBCR that can guide interventions to improve clinical outcomes in low- and middle-income countries.

## INTRODUCTION

There are approximately 200,000 new cases of childhood cancer diagnosed annually worldwide.^1,2^ In contrast to the current 5-year overall survival (OS) of 77% to 83% for children with cancer in high-income countries,^3-5^ outcomes in low- and middle-income countries (LMICs) show substantial disparities, with 5-year OS rates ranging of 5% to 60%.^6-12^

In Colombia, a middle-income country, an increasing trend in the proportion of cancer deaths in children was observed in the period 1985 to 2008.^3^ Recognizing the importance of assessing clinical outcomes in children with cancer to guide improvements in health care systems, a group of pediatric oncologists collaborated with Cali’s population-based cancer registry (PBCR)^13^ in 2008 to establish an integrated, real-time childhood cancer–specific outcomes surveillance system, VIGICANCER. The project’s aim was to gain a better understanding of factors associated with mortality in children with cancer in Cali and to use the knowledge gained to advocate for effective health policy interventions. Herein, we describe the implementation of VIGICANCER to collect survival data and assess outcomes of pediatric patients with cancer treated in Cali between 2009 and 2013.

## METHODS

### Setting

#### Demographics of Colombia and Cali.

During 2009 to 2013, Colombia had a population of 47.8 million and a per capita gross national income of US $5,090 to $7,970.^14^ The poverty rate (28.5%) remains higher than the Latin American average, and the 2013 Gini index (53.9) was the second highest in the region.^14-16^

The Valle province in Colombia has approximately 4.5 million inhabitants, including 1 million children.^17,18^ Its capital, Cali, the third most populous city in Colombia, had 550,171 children (< 15 years) and 185,705 adolescents (15 to 18.9 years).^17,19^ Of them, 23.1% were below the poverty line.^17,19,20^ The age-standardized incidence rate of childhood cancer in Cali during the study period was 141.2 cases per 1 million person-years.^9^

#### Health care system in Colombia and Cali.

In 1993, Colombia established a compulsory universal health insurance system.^21,22^ The system was designed to cover most of the population through two policies: the contributory plan (Spanish acronym POSc, Plan Obligatorio de Salud Contributivo), covered approximately 40% of the population, and the subsidized plan (Spanish acronym POSs, Plan Obligatorio de Salud Subvencionado), covered approximately 48% of the population. Approximately 12% of the population fell outside these two groups: 2% had private insurance in addition to POSc, 4% had government insurance (police, military, or government employees), and 6% were uninsured (PPNA, Poblacion Pobre No Asegurada). Cali’s health care system serves not only its inhabitants but also 41 other municipalities in the Valle province. In addition, patients with cancer were also referred to Cali from neighboring provinces.

#### Pediatric oncology units in Cali.

From 2009 to 2013, Cali had two major pediatric oncology units (POUs). The larger unit was located at Hospital Universitario del Valle, a public hospital, which served 45% of all pediatric patients with cancer in Cali. The other major POU was located at Fundación Valle del Lili, a private hospital, where 32% of pediatric patients with cancer were served. The remaining patients were served collectively by five POUs at private hospitals. Cali had five pediatric oncologists in 2009, and that number increased to eight in December 2013.

#### PBCR in Cali.

Cali’s PBCR was established in 1962 at the Universidad del Valle, the largest academic institution in the region. Cali’s PBRC has consistently and accurately reported incidence and mortality data for all pediatric and adult patients with cancer since its inception.^13,23^ In September 2012, the International Agency for Research on Cancer recognized Cali’s PBCR as a reliable data source for all 10 volumes of the monograph series *Cancer Incidence in Five Continents*.^24^

### Planning and Implementing VIGICANCER

#### Purpose.

VIGICANCER was developed as a sustainable method of systematic data collection to allow comprehensive and timely availability of data on clinical outcomes of all children and adolescents treated for cancer in Cali. The goal was to identify key determinants of survival that would provide the basis for developing interventions to improve long-term clinical outcomes.

The design of VIGICANCER was unique: it combined the PBCR, which aimed to collect epidemiologic information within a geographic region, with a hospital-based cancer registry (HBCR), which focused on collecting clinical data. This collaboration also ensured that data management was closely supervised by experienced PBCR staff. VIGICANCER was approved by the institutional review boards of Universidad del Valle and all participating POUs. Informed consent was obtained from parents of children participating in the study. Since the start of the project, only one parent has declined participation.

#### Working group.

An interdisciplinary working group consisting of medical and other allied professionals was formed. The group comprised four pediatric oncologists, one pediatric oncologist-epidemiologist, one pathologist-epidemiologist, five PBCR data managers, three VIGICANCER clinical monitors, one informatics engineer, and one administrator. A senior clinical monitor was appointed as leader to supervise the training of and data collection by other clinical monitors. The group was trained and supervised through courses, site visits by the senior clinical monitor, web-based meetings, telephone calls, and encrypted e-mails to ensure accurate data collection.

#### Quality assurance.

Five main indicators of quality were measured: number of patients included per month (goal, ≥ 15), proportion of patients actively detected for follow-up (goal, > 90%), proportion of patients included but lost to follow-up (goal, < 10%), proportion of deaths detected by the system (goal, > 95%), and number of scientific communications released to health authorities and the general public each year (goal, one or more). In addition, quality checks were conducted to ensure that missing information on key variables was < 10%, detect and correct misclassification errors in real time, and minimize cases that were not pathologically confirmed to 1% to 5%.

#### Long-term sustainability.

Start-up funds for VIGICANCER were provided by the Sanofi-Espoir Foundation through the My Child Matters program,^25^ which aims to improve childhood cancer care in LMICs and reduce inequalities in outcomes of children between LMICs and HICs. In 2010, the local nongovernmental foundation Pediatric Oncologist and Hematologist (POHEMA) was created by pediatric oncologists in Cali to ensure sustainability by generating funds to operate VIGICANCER

### Study Population, Measurements, and Data Collection and Analysis

#### Case definition and identification.

VIGICANCER included patients younger than19 years of age, registered at any of the five POUs in Cali, with a new diagnosis of a malignant neoplasm. Patients with benign lesions of the CNS, except for craniopharyngiomas, were also included. Patients treated with only surgery outside a POU were subsequently detected and registered by Cali’s PBCR. Patients who died before being referred to a POU were also registered by Cali’s PBCR.

In the absence of a pathology report, which is the gold standard for cancer diagnosis, clinical criteria as well as imaging and supporting laboratory tests (if applicable) were used to confirm diagnosis. Some cases were detected solely by death certificates. If a patient newly identified through a pathology report was not under treatment at any POU, only basic epidemiologic information was noted and the case was reported to Cali’s PBCR.

#### Demographic and baseline clinical information.

The following baseline demographic variables were collected: date of birth, sex, race/ethnicity, place of residence, place of diagnosis (POU, pathology laboratory, or other), type of health insurance, and whether care transferred to another institution. The latter happened in approximately 25% of pediatric patients with cancer dictated by insurance contracts with POUs. The following baseline clinical variables were collected: pathologic diagnosis, tumor location, date of diagnosis, and method of diagnosis. The International Classification of Diseases for Oncology, 3rd edition (ICD-O-3) was used for disease coding.^26^ For hematologic and lymphoid neoplasms, the WHO updated version of ICD-O-3 was used.^27^ The International Childhood Cancer Classification, 3rd edition (ICCC-3) was used for basic analyses.^28^ A simplified staging classification was used, where applicable, to classify tumors as localized, regional, or metastatic. For each tumor group, information on some additional predictors of outcome (Table 1) was also obtained.

**Table 1:**
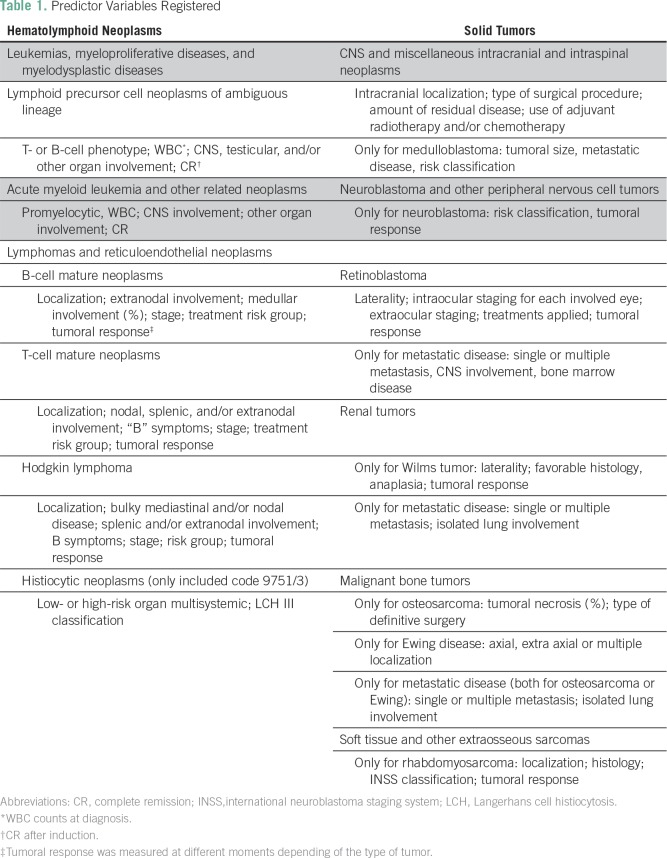
Predictor Variables Registered

#### Outcomes.

The primary outcome was death. Secondary outcome variables were events such as relapse, progression, second malignancy, and treatment abandonment. Relapse was defined as histopathologic evidence of disease recurrence after previously documented complete remission. Diagnosis of relapse was made without pathologic confirmation in cases where unequivocal evidence of relapse was available on imaging and/or positive tumor markers. Disease progression was defined as ≥ 25% increase in two-dimensional measurements of the visible tumor(s) on imaging. Second malignancy was defined as a histologically distinct second cancer developing after the first cancer. Treatment abandonment was defined as an unplanned delay in therapy for > 4 weeks that was unrelated to medical reasons and due to the patient not returning for care.^29^ All cases of treatment abandonment were included in VIGICANCER. Efforts to contact patients abandoning treatment (eg, telephone calls to families) were made to maintain surveillance and collect vital status information. Treatment abandonment was considered an event for survival analyses and documented in the same manner as no subsequent relapse or death.

#### Follow-up.

Clinical monitors gathered information every 3 months from medical records or by contacting families to document disease status as active or in remission and information on change of POU. Lost to follow-up was defined as a patient missing a scheduled appointment without informing, without medical justification during active therapy (treatment abandonment), or after finishing therapy, and being noncontactable by any means for 6 months despite at least three phone contact attempts. After loss to follow-up was documented, passive surveillance was continued through Cali’s PBCR to determine vital status.

#### Data sources.

**Fig 1:**
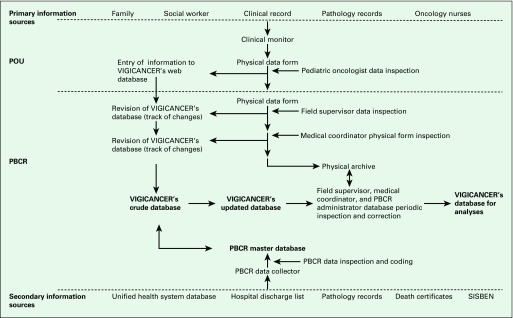
Information sources and flow for VIGICANCER. PBCR, population-based cancer registry; POU, pediatric oncology unit; SISBEN, Identification System for Potential Beneficiaries of Social Programs.

Active surveillance for new case identification was conducted in each of Cali’s POUs (Fig 1). However, in 2013, 10.3% of patients were identified from secondary sources used by Cali’s PBCR, especially for patients with solid tumors who received only surgical treatment. Secondary sources included hospital logs for patient discharges, pathology laboratories not located in POUs, the death record database at the Municipal Health Department in Cali, and the National Health Insurance database. In 1% of cases, death certificate was the exclusive source of case identification.

**Fig 2:**
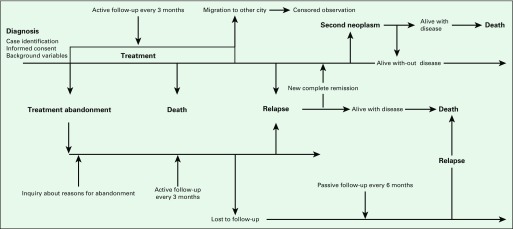
Patient follow-up and main events identified by VIGICANCER.

Relevant information on background variables was actively collected from patients’ medical records, pathology reports, and notes of social workers at POUs. Where needed, additional information was acquired directly from patients’ caregivers (Fig 2). Clinical updates were taken directly from treating oncologists. Clinical monitors liaised closely with oncologists, patients, and their families to obtain any missing information. Events such as death, relapse, disease progression, second malignancy, and treatment abandonment were all recorded.

#### Statistical analyses.

Statistical analyses were conducted using STATA (version 13.1; StataCorp, College Station, TX). Central tendency and dispersion measures were used for continuous variables. Absolute and relative frequencies were calculated for categorical variables. Cross-tabulations were constructed according to the ICCC-3, and each demographic and clinical characteristic was measured.

Event-free survival (EFS) was calculated from the date of diagnosis to the date of first event. OS was calculated from the date of diagnosis to the date of death or treatment abandonment if no vital status was known after the event. Data on patients detected only by death certificate (without diagnosis date) were excluded from survival analyses, because time to event could not be estimated. The last time point to follow-up was documented for patients who were alive with no events and was considered as a censored observation. No case of treatment abandonment was included in the analyses as a censored observation. Patients with change in address or lost to follow-up were censored when no treatment abandonment was documented. Kaplan-Meier survival curves were generated for OS and EFS from these survival probabilities, and 95% CIs were calculated.

## RESULTS

We report the analysis of patients diagnosed from January 1, 2009 to December 31, 2013. The last date to follow-up for this cohort was December 31, 2015.

### Demographics and Diagnoses

**Fig 3:**
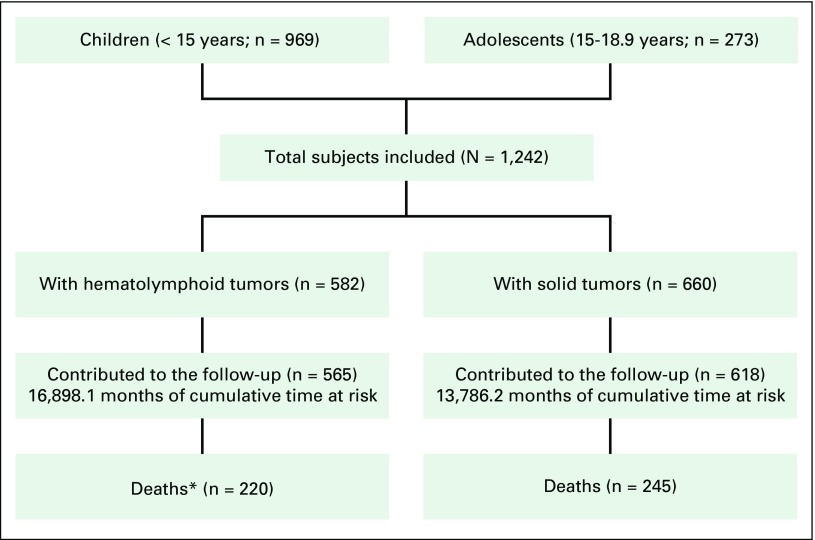
Flow diagram of patients included in the system (2009 to 2013). (*)Includes patients with treatment abandonment without information about vital status in the follow-up.

Figure 3 presents a flowchart of patients included in VIGICANCER. Data showed that 32.4% of patients (95% CI, 29.8% to 35.0%) were younger than 5 years, 55.0% (95% CI, 53.3% to 58.8%) were male, and 15.6% (95% CI, 13.3% to 18.1%) were Afro-descendants. Overall, the frequency of ICCC group I tumors was high in all age groups (Table 2) except for children younger than 1 year old, in whom CNS tumors were more frequent (26.6%; 95% CI, 17.0% to 39.0%). In children (< 15 years of age), the major ICCC tumor groups were I (37.3%; 95% CI, 34.5% to 40.6%), III (18.8%; 95% CI, 16.4% to 21.4%), and II (11.1%; 95% CI, 9.1% to 13.1%). Children residing outside of Cali were more likely to have POSs (51.6% *v* 30.1%, respectively; *P* < .01). This observation was similar among age groups, sex, race/ethnicity, and period of study enrollment.

**Table 2:**
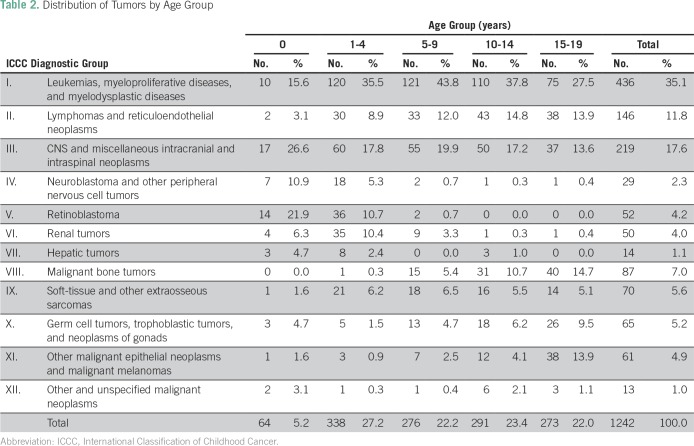
Distribution of Tumors by Age Group

### Clinical Outcomes

As of December 31, 2015, 37.4% (95% CI, 34.8% to 40.2%) of patients have died (Fig 3). One percent (95% CI, 0.7% to 2.1%) died on the same day of diagnosis, and 2.1% (95% CI, 1.4% to 3.1%) died 1 to 5 days after diagnosis. Table 3 shows OS of children by ICCC groups.

**Table 3:**
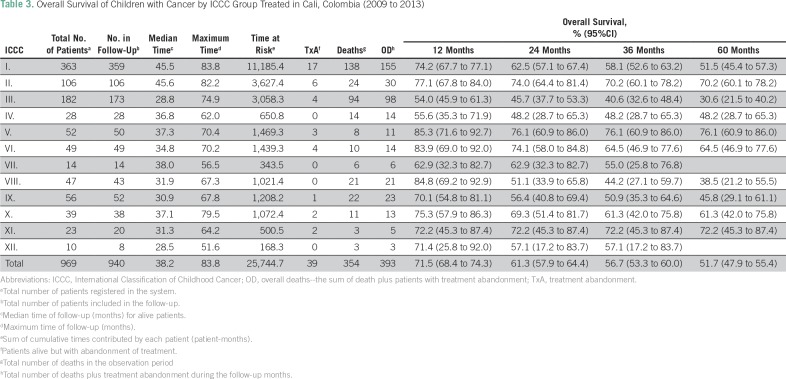
Overall Survival of Children with Cancer by ICCC Group Treated in Cali, Colombia (2009 to 2013)

Five-year OS for all cancers was 51.7% (95% CI, 47.9% to 55.4%) for children and 39.4% (95% CI, 29.8% to 50.5%) for adolescents (15 to 18.9 years). Five-year OS and EFS for acute lymphoblastic leukemia (ALL) was 55.6% (95% CI, 48.5% to 62.2%) and 48.7% (95% CI, 41.9% to 55.0%), respectively.

Of 281 children with ALL, death during induction treatment occurred in 5.3% (95% CI, 3.2% to 8.7%), death due to disease progression/relapse occurred in 14.2% (95% CI, 10.6% to 18.9%), death was not attributable to disease in 10.0% (95% CI, 7.0% to 14.1%), and cause of death was not classified in 2.5% (95% CI, 1.2% to 5.2%) of patients. Cumulative incidence of treatment abandonment for all children at 24 months was 12.2% (95% CI, 9.8% to 15.2%) and for children with ALL was 16.0% (95% CI, 11.4% to 22.3%).

## DISCUSSION

Survival estimates of children with cancer obtained from HBCRs are mainly used in clinical trials to document treatment effectiveness at the participating institution(s). However, intrinsic selection bias can preclude generalization of these estimates. Although HBCRs usually overestimate actual population survival probabilities, they allow the collection of complex subsets of clinical data, which is usually not feasible with PBCRs.

VIGICANCER is unique because it combines the strengths of both PBCRs and HBCRs. It is an outcomes surveillance system for data on childhood cancers embedded within a PBCR, but it directly collects information from treating POUs. We show that VIGICANCER can successfully document relevant clinical events for patients with childhood cancer receiving treatment within its catchment area in an acceptable time frame. It yields reliable outcomes, good follow-up, and a high level of data completeness for independent variables.

Our system was based on four principles: exhaustiveness—ability to represent all pediatric cancers treated in the catchment area; timeliness—ability to collect real-time data; simplicity—ability to collect minimal but most relevant information; and observation—based on ongoing clinical care, thereby reflecting real-life practice.

Because childhood cancers are generally not preventable, survival is a more relevant metric than incidence to measure the success of health policy efforts. OS reflects the effectiveness of cancer-related health services, and PBCRs rely mainly on this parameter.^11^ Despite its utility, measuring only OS as outcome is not reliable enough to plan effective interventions, because various causes can underlie poor outcomes in patients with cancer. The key to understanding prevailing conditions is to document all possible outcomes as events. In LMICs, treatment abandonment is a major factor for disease relapse, therefore making it an important variable to monitor.^30^ PBCRs do not yield reliable data about variables such as treatment abandonment and relapse. For example, according to Cali’s PBCR data for 2002 to 2006, the 5-year OS estimate for children living in Cali was 54.9%, using PBCR data for the period 2002 to 2006.^9^ When compared with the 5-year OS rate of 51.7% in our study, it seems that OS for pediatric patients with cancer has not improved in the last 10 years. However, on closer analysis of the estimate derived from Cali’s PBCR data, all patients lost to follow-up were censored observations, which led to overestimation of OS. Thus, having a dedicated cancer outcomes surveillance system is the most reliable way to collect detailed information about all relevant events.

There were several advantages of embedding VIGICANCER within a PBCR. First, Cali’s PBCR was already recognized and respected as an independent and accurate source of cancer data in the region, which ensured cooperation from all POUs and public health officials in Cali. Second, we were able to reduce information bias and missing data—especially for deaths occurring at home—and possible patients treated in Cali but not registered in VIGICANCER, by ultimately identifying such cases by the PBCR, which uses alternative sources of information. These crucial aspects essential to achieve valid population-based estimates cannot be obtained from HBCRs alone. Moreover, crossover with the PBCR allowed the identification of patients who died untreated, which is particularly relevant in LMICs, where access to pediatric oncology centers is delayed after diagnosis.

Conversely, most LMICs lack a preexisting PBCR^31^; therefore, our model of VIGICANCER embedded within a PBRC may present implementation challenges in LMICs. Compared with HBCRs, VIGICANCER is more complex, labor intensive, and expensive to establish. However, our experience shows that the biggest challenge is to establish effective collaboration among local experts, which is an essential part of successful implementation.

VIGICANCER has been critical in establishing the baseline status of outcomes of children with cancer in Cali. In LMICs with similar settings, this model can be replicated to evaluate the burden of childhood cancer and identify locally relevant focus areas to improve patient outcomes.

In conclusion, VIGICANCER is a surveillance system implemented in an LMIC that, to our knowledge, is the only system that can be embedded in a PBCR. It has accurately established the baseline status of pediatric cancer outcomes in Cali and most likely causes of treatment failure. VIGICANCER not only yields real-time accurate and meaningful epidemiologic and clinical data within its catchment area but also strengthens pediatric oncology centers and local health care systems in a sustainable manner by allowing immediate application of useful knowledge to improve outcomes of underserved populations. We plan to extend its implementation to the southwestern region of Colombia and ultimately to neighboring regions. Our experience shows that it is feasible to implement a comprehensive childhood cancer surveillance system in resource-limited settings, provided there is political will and professional motivation to collaborate.

## References

[1] Kellie SJ, Howard SC (2008). Global child health priorities: What role for paediatric oncologists?. Eur J Cancer.

[2] Rodriguez-Galindo C, Friedrich P, Morrissey L (2013). Global challenges in pediatric oncology. Curr Opin Pediatr.

[3] Gatta G, Botta L, Rossi S (2014). Childhood cancer survival in Europe 1999-2007: Results of EUROCARE-5--A population-based study. Lancet Oncol.

[4] Ward E, DeSantis C, Robbins A (2014). Childhood and adolescent cancer statistics, 2014. CA Cancer J Clin.

[5] Smith MA, Altekruse SF, Adamson PC (2014). Declining childhood and adolescent cancer mortality. Cancer.

[6] Piñeros M, Gamboa O, Suárez A (2011). Mortalidad por cáncer infantil en Colombia durante 1985 a 2008. Rev Panam Salud Publica.

[7] Moreno F, Dussel V, Orellana L (2015). Childhood cancer in Argentina: 2000-2007. Cancer Epidemiol.

[8] Valsecchi MG, Tognoni G, Bonilla M (2004). Clinical epidemiology of childhood cancer in Central America and Caribbean countries. Ann Oncol.

[9] Bravo LE, García LS, Collazos P (2013). Descriptive epidemiology of childhood cancer in Cali: Colombia 1977-2011. Colomb Med (Cali).

[10] Pacheco C, Lucchini G, Valsecchi MG (2014). Childhood acute lymphoblastic leukemia in Nicaragua: Long-term results in the context of an international cooperative program. Pediatr Blood Cancer.

[11] Allemani C, Weir HK, Carreira H (2015). Global surveillance of cancer survival 1995-2009: Analysis of individual data for 25,676,887 patients from 279 population-based registries in 67 countries (CONCORD-2). Lancet.

[12] Ribeiro RC, Steliarova-Foucher E, Magrath I (2008). Baseline status of paediatric oncology care in ten low-income or mid-income countries receiving My Child Matters support: A descriptive study. Lancet Oncol.

[13] Universidad del Valle Registro Poblacional de Cáncer de Cali..

[14] The World Bank Data: Columbia.

[15] The World Bank World Development Indicators: Health Systems.

[16] The World Bank (2015). Documents and Reports: Colombia - Systematic country diagnostic.

[17] Colombian National Statistics Department (DANE) https://www.dane.gov.co/index.php/estadisticas-por-tema/demografia-y-poblacion/censo-general-2005-1.

[18] World Heritage Encyclopedia Valle del Cauca Department.

[19] (2009).

[20] Colombian National Statistics Department (DANE) Pobreza y desigualdad..

[21] Montenegro T, Acevedo F, Bernal O (2013). Colombia Case Study: The Subsidized Regime of Colombia’s National Health Insurance System. Universal Health Coverage (UNICO) studies series; no. 15. Report Number 74961.

[22] Giedion U, Villar-Uribe M (2009). Colombia’s Universal health insurance system: The results of providing health insurance for all in a middle-income country. Health Aff (Millwood).

[23] Cendales R, Pardo C, Uribe C (2012). Data quality at population-based cancer registries in Colombia. Biomedica.

[24] Forman D, Bray F, Brewster DH (eds) (2014). Cancer Incidence in Five Continents, Vol. X. IARC Scientific Publication 1o. 164.

[25] Burton A (2009). The My Child Matters awards: New funding, new countries, new hope. Lancet Oncol.

[26] World Health Organization (2013). International Classification of Diseases for Oncology.

[27] Swerdlow SH, Campo E, Harris NL (2008). WHO Classification of Tumours of Haematopoietic and Lymphoid Tissues.

[28] Steliarova-Foucher E, Stiller C, Lacour B (2005). International Classification of Childhood Cancer, third edition. Cancer.

[29] Mostert S, Arora RS, Arreola M (2011). Abandonment of treatment for childhood cancer: Position statement of a SIOP PODC Working Group. Lancet Oncol.

[30] Arora RS, Eden T, Pizer B (2007). The problem of treatment abandonment in children from developing countries with cancer. Pediatr Blood Cancer.

[31] Magrath I, Steliarova-Foucher E, Epelman S (2013). Paediatric cancer in low-income and middle-income countries. Lancet Oncol.

